# *In planta* production of ELPylated spidroin-based proteins results in non-cytotoxic biopolymers

**DOI:** 10.1186/s12896-015-0123-2

**Published:** 2015-02-19

**Authors:** Valeska Hauptmann, Matthias Menzel, Nicola Weichert, Kerstin Reimers, Uwe Spohn, Udo Conrad

**Affiliations:** Leibniz Institute of Plant Genetics and Crop Plant Research, Corrensstrasse 3, 06466 Stadt Seeland, OT Gatersleben, Germany; Fraunhofer Institute for Mechanics of Materials, Walter-Hülse-Strasse 1, 06120 Halle/Saale, Germany; Department of Plastic, Hand and Reconstructive Surgery, Hannover Medical School, Podbielskistr. 380, 30659 Hannover, Germany

**Keywords:** Spider silk, Immunogenicity, Synthetic spidroin, Biomaterial, Biocompatibility, Atomic force microscopy

## Abstract

**Background:**

Spider silk is a tear-resistant and elastic biopolymer that has outstanding mechanical properties. Additionally, exiguous immunogenicity is anticipated for spider silks. Therefore, spider silk represents a potential ideal biomaterial for medical applications. All known spider silk proteins, so-called spidroins, reveal a composite nature of silk-specific units, allowing the recombinant production of individual and combined segments.

**Results:**

In this report, a miniaturized spidroin gene, named *VSO1* that contains repetitive motifs of *MaSp1* has been synthesized and combined to form multimers of distinct lengths, which were heterologously expressed as elastin-like peptide (ELP) fusion proteins in tobacco. The elastic penetration moduli of layered proteins were analyzed for different spidroin-based biopolymers. Moreover, we present the first immunological analysis of synthetic spidroin-based biopolymers. Characterization of the binding behavior of the sera after immunization by competitive ELISA suggested that the humoral immune response is mainly directed against the fusion partner ELP. In addition, cytocompatibility studies with murine embryonic fibroblasts indicated that recombinant spidroin-based biopolymers, in solution or as coated proteins, are well tolerated.

**Conclusion:**

The results show that spidroin-based biopolymers can induce humoral immune responses that are dependent on the fusion partner and the overall protein structure. Furthermore, cytocompatibility assays gave no indication of spidroin-derived cytotoxicity, suggesting that recombinant produced biopolymers composed of spider silk-like repetitive elements are suitable for biomedical applications.

**Electronic supplementary material:**

The online version of this article (doi:10.1186/s12896-015-0123-2) contains supplementary material, which is available to authorized users.

## Background

Recent biomedical developments in the field of tissue engineering require protein-based biomaterials as scaffolds that have been optimized for substantial extensibility, long-term stability, self-assembly and low energy loss [1]. Further important properties are biocompatibility and economical production, as well as purification systems, which could be provided by plant-based production of elastin-like peptide (ELP) fusion proteins, referred to as ELPylated proteins [2,3]. Since ancient times, spider silk has been known for its extraordinary properties. Bygone cultures used the secretory product of the spider spinning glands. For example, Ancient Greeks were aware of the boosting effect for wound healing and used spider webs to cover bleeding lesions [4]. Today, spider silk is known to promote the regeneration of nerves [5], and supports the proliferation of fibroblasts and keratinocyte cell lines [6]. In addition to these fascinating features, the lightweight and flexible biopolymer spider silk joins mechanical properties such as high toughness, tensile strength and stiffness that competes with man-made polymers [7].

Spider silks produced by orb-web-weaving spiders are composed of proteins that are commonly termed spidroins. An intensively investigated silk is the major ampullate silk, which is used by spiders as a structural element to build the web frame and the safety line; therefore, it is also called dragline silk. The well-known structure of major ampullate silk consists of two proteins called major ampullate spidroin 1 (MaSp1) and major ampullate spidroin 2 (MaSp2) [8]. The first partial sequence information from MaSp1 of *Nephila clavipes* was published in 1990 and revealed a high repetitive primary structure [9]. The protein consists of poly(A) blocks alternating with GGX (X = Y, L, Q) and (GA)_n_ sequence motifs [10]. Currently, the high number of repetitive peptide motifs in the core sequence is known as the key feature of spidroins. Clearly there is a strong relationship between the secondary structure, based on the unique motifs in the primary protein structure, and the outstanding properties of spider silks. The alanine-rich peptide regions form a β-sheet that provides remarkable strength to major ampullate silk [11]. Conversely, the high toughness arises from the glycine-rich peptide motifs, which likely induce the formation of β-turns and 3_10_ helices [7,12]. For the spidroin MaSp1, a molecular weight of up to 320 kDa is reported [13]. It is assumed that the size of spider silk proteins is a key factor in defining their mechanical properties, because all characterized native spider silks consist of proteins with high molecular weights [13].

The usage of native spider silk on a larger scale is not economically profitable. Currently, heterologous spidroin production is the method of choice to satisfy the demand of recombinant spider silk for research. For this purpose, the most widely used host system is the gram-negative bacterium *Escherichia coli*. Here, spider silk protein modules of mainly a low molecular weight, approximately 50 kDa, were produced [14-17]. However, heterologous expression of recombinant spider silk proteins in *E. coli* was found to be rather inefficient owing to the low production rate and instability of the spider silk gene [18]. Because of the highly repetitive nature of the proteins, DNA deletion in the spider silk gene, as well as transcription and translation errors were often observed during the reproduction of recombinant *E. coli* harboring the gene [19]. Furthermore, translational errors of proteins were caused by a depletion of the t-RNA pool owing to the high alanine and glycine content. Recently, expression of high molecular weight spider silk derivatives up to 285 kDa has been achieved by optimizing the glycyl-tRNA amount and glycine synthesis [20]. Other expression systems used for heterologous spidroin production are yeast [21], plants [22,23], insects [24] or mammalians [25].

The knowledge of the molecular structure of spider silk has inspired researchers to use the repeated modules of silks to develop synthetic spidroins. In addition, an approach using a synthetic gene can avoid the abovementioned difficulties during heterologous expression of spider silk proteins. The adaption of the codon usage to the t-RNA pool of the intended host system is a considerable advantage of synthetic genes. Furthermore, restriction sites necessary for cloning into expression vectors can be attached during gene synthesis and, therefore, prevent additional PCR reactions that often cause errors with highly repetitive genes. In previous studies, the synthetic spider silk protein SO1, which shows a 94% homology to MaSp1 of the golden silk spider, was successfully expressed in plants *(Nicotiana tabacum, Solanum tuberosum*) [22]. For further investigations, this synthetic spidroin was fused to 100 repeats of ELP to facilitate the purification of plant-produced spider silk-like proteins [26]. In addition, for other spider silk proteins ELPylation is a powerful technology for easy and economical purification [27].

In general, low immunogenicity is anticipated for highly repetitive proteins such as spider silk derivatives and ELP [28]. Here, we designed various synthetic spidroin-based fusion proteins consisting of several repetitive motifs of SO1 and accordingly MaSp1 termed (VSO1)_n_-100xELP. We have designed these artificial fusion proteins to produce material for tissue engineering with suitable mechanical properties, low immunogenicity and cytocompatibility. After heterologous expression in plants, the spidroin-based fusion proteins were purified by a scalable and economical downstream processing procedure. Furthermore, these synthetic spidroins were used to analyze their mechanical properties and immunogenicity. By producing recombinant spider silks in different formats, the main requirements for biomedical applications, including biocompatibility, sufficient mechanical properties in terms of elasticity, hardness and stiffness, and low or even no immunogenicity, are met. Biocompatibility is defined as the quality of the biomaterial as not being toxic or having injurious effects on biological systems [19]. In the present paper we have measured the cytocompatibility with murine embryonic fibroblasts as an approach to estimate biocompatibility. Finally, we discuss the suitability of the different recombinant synthetic spider silk proteins for biomedical applications.

## Results

### Recombinant production of synthetic spidroin-based fusion proteins

**Figure 1:**
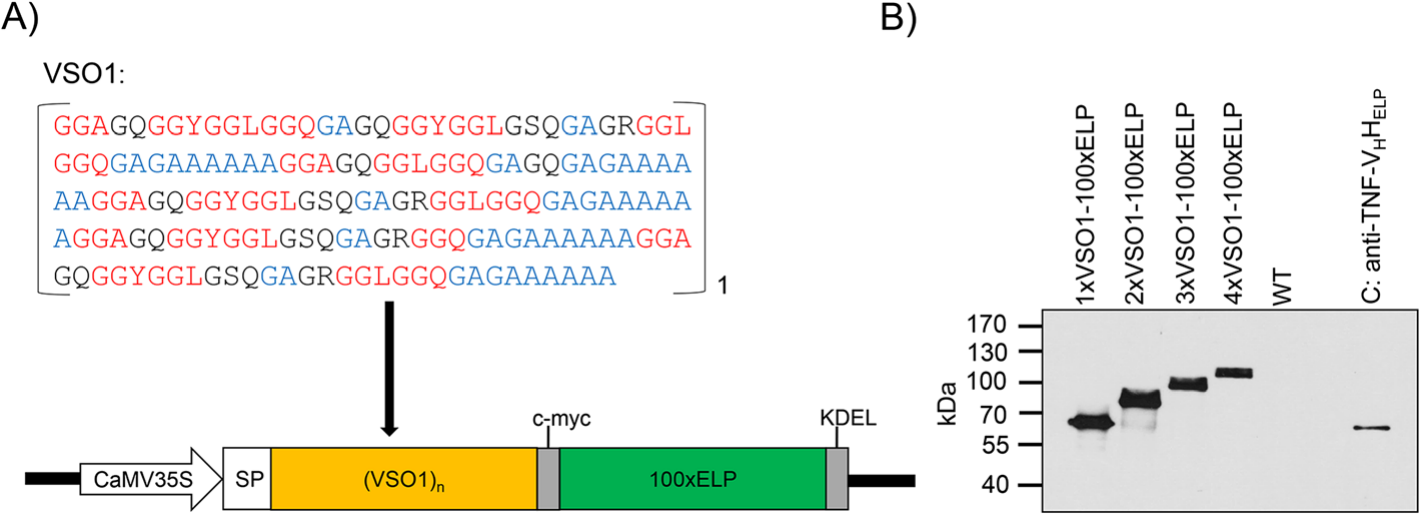
**Expression of spidroin-based fusion proteins (VSO1)**_**n**_ **-100xELP in transgenic tobacco plants. A)** Protein sequence of VSO1 and schematic representation of the plant expression cassette for (VSO1)_n_-100 × ELP. In the protein sequence, repetitive motifs are highlighted in red and blue. Heterologous expression of synthetic spidroin (VSO1)_n_ fused to 100 repeats of elastin-like peptide (100xELP) was controlled by the CaMV 35S promoter. SP: legumin B4 signal peptide; KDEL: ER retention signal; c-myc: detection tag. **B)** Immunoblot of leaf extracts from transgenic tobacco expressing spider silk fusion proteins of different sizes. Samples of 5 μg of total soluble protein were applied to the gel and separated by 8% SDS-PAGE, blotted and detected with an anti-c-myc antibody. WT: wild type *N. tabacum* cv. SNN. C+: 1 ng c-myc immunoblot standard anti-TNF-V_H_H-100xELP.

Four different plant expression vectors coding for spidroin-based fusion proteins varying in the spidroin content have been designed to assess the mechanical properties, immunogenicity and cytotoxicity in relation to the protein size. Therefore, we created the synthetic spidroin gene *VSO1* containing characteristic repetitive motifs of *MaSp1* and *SO1*, respectively, which were used as model proteins for the blueprint. The synthesized gene was flanked by compatible but non-regenerative restriction sites (*Bam*HI and *Bgl*II) to enable the insertion of further synthetic genes for extension of the spidroin content. The resulting recombinant proteins contained the typical sequence motifs GGX, (GA)_n_ and polyA blocks of MaSp1 in the primary sequence (Figure 1A). Additionally, the synthetic spidroin genes were combined with the gene encoding for the biopolymer 100xELP in an expression vector to facilitate ubiquitous expression in transgenic tobacco. For the retention of the recombinant proteins in the endoplasmic reticulum (ER), the expression cassette also contained the signal peptide (SP) LeB4 [29] and the ER retention signal KDEL [30] (Figure 1A). Thus, four different synthetic spidroin-based biopolymers were designed with calculated molecular weights of 56 kDa (1xVSO1-100xELP), 69 kDa (2xVSO1-100xELP), 83 kDa (3xVSO1-100xELP) and 96 kDa (4xVSO1-100xELP). Initially, each expression vector was individually transformed into *Agrobacterium tumefaciens* (*A. tumefaciens*), followed by the generation of transgenic tobacco (*Nicotiana tabacum*) by leaf disc transformation. After selection of kanamycin-resistant tobacco plants, expression of fusion proteins was proven by immunoblotting. Analyzed leaf extracts showed the appropriate size increase from 1xVSO1-100xELP to 4xVSO1-100xELP (Figure 1B). The molecular weights were determined by SDS-PAGE and, accordingly, immunoblotting. Protein sizes appeared higher than the calculated masses, a phenomenon that was also observed for other ELP fusion proteins [31,32].

The principal reason for fusion of the synthetic spidroins to 100 repeats of ELP was to enable the chromatography-free purification by inverse transition cycling (ITC) [26,33], a low cost method for recovery of biopolymers. Here, synthetic spidroin-based fusion proteins were purified from tobacco leaves of transgenic plants *via* an advanced membrane based ITC [34], which was optimized for spider silks and performed according to Weichert *et al*. [27]. A heat incubation step at the beginning of the purification procedure leads to denaturation of the majority of the proteins. In the following cooling step (4°C), all fusion proteins became soluble and were separated by centrifugation and enriched by filtration after several temperature shifts. For further investigations purified proteins were analyzed as casted layers or in solution.

### Mechanical investigation of synthetic spidroin-based biopolymer layers

**Figure 2:**
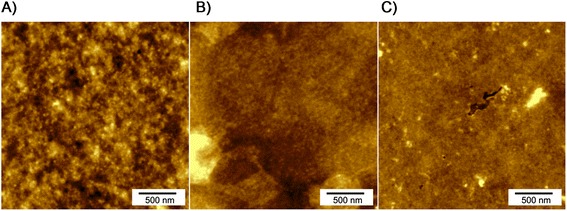
**AFM topographic images of spidroin-based biopolymer layers.** Surfaces have been taken in the intermitting tapping mode using 2.5 × 2.5 μm^2^ scan sizes of **A)** 1xVSO1-100xELP, **B)** 2xVSO1-100xELP and **C)** 4xVSO1-100xELP.

Based on freshly prepared and smooth layers of the synthetic biopolymers 1xVSO1-100xELP, 2xVSO1-100xELP and 4xVSO1-100xELP, we performed Atomic Force Microscopy (AFM) based nanoindentation experiments to examine the relevance of the increasing spidroin content. Figure 2 shows the corresponding topographic images with the z-ranges of 9.6 nm (1xVSO1-100xELP), 7.4 nm (2xVSO1-100xELP) and 11.4 nm (4xVSO1-100xELP), respectively. All surfaces appeared homogenous with amorphous regions. It should be emphasized here that the roughness of the examined layers is very low, with roughness values < 2 nm (Table 1), fulfilling the presumption for AFM-based nanoindentation to examine the elastic penetration modulus E and therefore, the characterization of the stiffness of the biopolymer layers. In former studies we showed on the basis of layers prepared from a monomeric tagged spider silk protein (MaSp1) fused to 100 repeats of ELP in comparison to layers consisting of multimers of this protein that the elastic penetration modulus E increased by multimerization [27]. In this mentioned experiment, the mass relation between the spider silk part and ELP remains unchanged during the multimerization process. In the present study, the determination of the elastic penetration modulus E of biopolymer layers varying in the spidroin content enabled a more detailed characterization of the relation between the molecular weight of the whole biopolymer and the spidroin content. Experiments were performed at thin layers of each casted layer with a thickness of at least 1 μm and a mean surface roughness smaller than 2 nm for 2.5 × 2.5 μm^2^ grids (Table 1). The E values presented in Table 2 are significantly different, as tested by the differential *t*-test [35]. It is important to notice that the E values of 2xVSO1-100xELP and 4xVSO1-100xELP were found to be considerably higher than this of 1xVSO1-100xELP. In comparison, AFM-based nanoindentation analyses of the fusion protein 100xELP revealed an E value of 2.74 GPa [27]. The differences between all mean values of the penetration modulus E are highly significant. It can be assumed that the increase of the spidroin content in the fusion proteins leads to an ascending E value, and therefore represents a higher stiffness of these materials.

**Table 1:** **Surface roughness of various synthetic biopolymer layers**

**Substance**	**Mean surface roughness s**_**a**_ **(nm)**	**Squared surface roughness s**_**q**_ **(nm)**
1xVSO1-100xELP	0.70	0.89
2xVSO1-100xELP	0.78	1.03
4xVSO1-100xELP	0.34	0.55

Defined grids of 2.5 × 2.5 μm^2^ were analyzed.

**Table 2:** **Elastic penetration moduli of recombinant spidroin-based fusion protein layers**

**Substance**	**Elastic penetration modulus E (GPa)**
1xVSO1-100xELP	2.73 ± 0.02
2xVSO1-100xELP	3.85 ± 0.02
4xVSO1-100xELP	4.51 ± 0.03
LDPE Riblene®FL30	0.172 ± 0.001

GPa: Gigapascal; ELP: elastin-like peptide.

Values were obtained by AFM-based nanoindentation under the following conditions: temperature = 23°C, relative humidity = 35%, system sensitivity = 22.5 nm/V, spring constant = 59.3 N/m, tip radius = 112 nm, setpoint control = 500 nN, relative setpoint = 2500 nN, z-closed loop active, z-length = 1 μm, retract delay = 10 ms, grid size = 25 × 25 μm^2^, data point 35 × 35 per run with an orthogonal and lateral inter-sampling point distance of at least 715 nm. Low density polyethylene (LDPE) Riblene® FL30 foil was used as reference.

To study in greater detail the influence of the increasing spidroin content in these ELPylated recombinant proteins, analyses of immunogenicity and cytotoxicity were performed and related to their potential use as biomaterials.

### Immunogenicity of synthetic spidroin-based fusion proteins 1xVSO1-100xELP and 4xVSO1-100xELP

**Figure 3:**
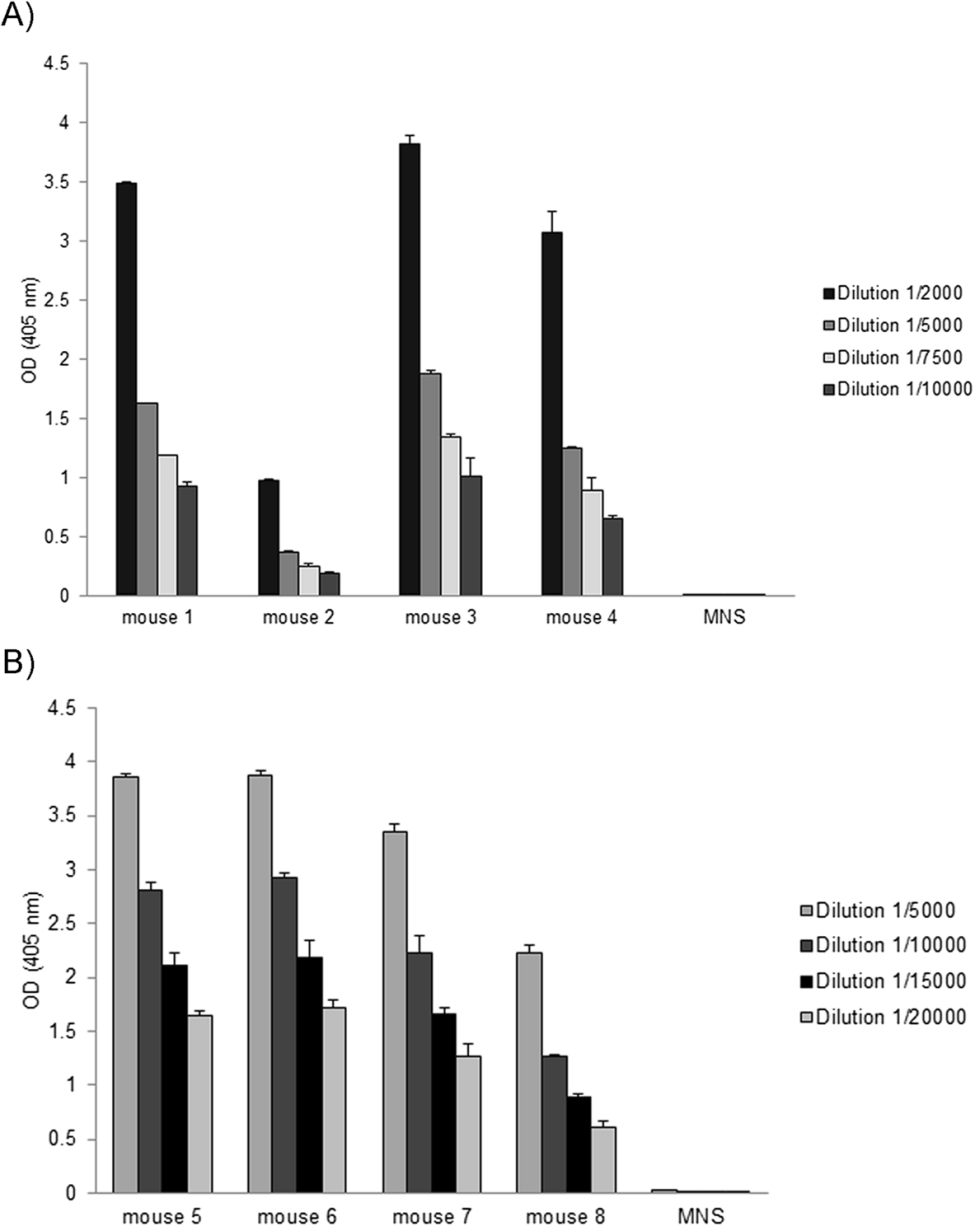
**Specific immune response of mouse sera determined by indirect ELISA.** Samples of 500 ng per well of **A)** 1xVSO1-100xELP and **B)** 4x VSO1-100xELP were coated to a microtiter plate and the reaction against mouse sera was analyzed for different dilutions. The antigen-antibody complex was detected using goat anti-mouse IgG conjugated with alkaline phosphatase. Extinction was measured at 405 nm (ordinate).

**Figure 4:**
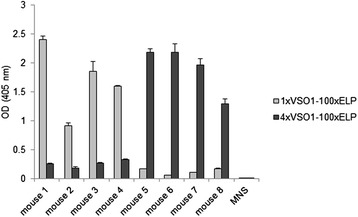
**Determination of the cross-reaction of mouse sera by indirect ELISA.** Antigens 1xVSO1-100xELP and 4xVSO1-100xELP were coated and the cross-reaction against the other antigen was analyzed. Mouse sera specific against 1xVSO1-100xELP were applied at a dilution of 1:2000 and mouse sera specific against 4xVSO1-100xELP were diluted at 1:5000. Extinction was measured at 405 nm (ordinate).

**Figure 5:**
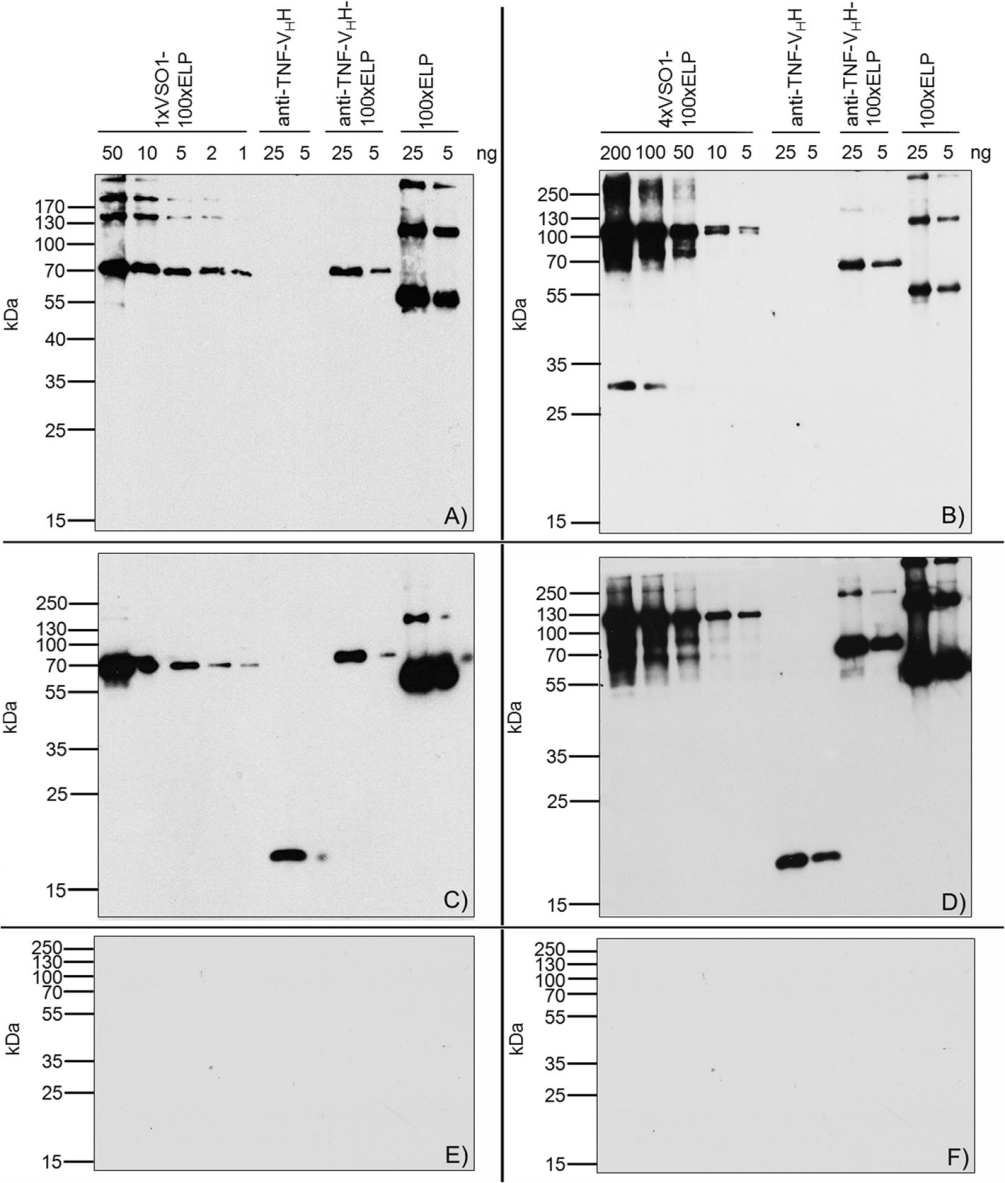
**Comparative immunoblots of the immune response of plant-expressed biopolymers to different antibodies.** Recombinant proteins were separated on 12% SDS-PAGE, electrotransferred and detected with **A)** mouse serum number 1, **B)** mouse serum number 5, **C)** and **D)** anti-c-myc monoclonal antibody, and **E)** and **F)** mouse normal serum.

**Figure 6:**
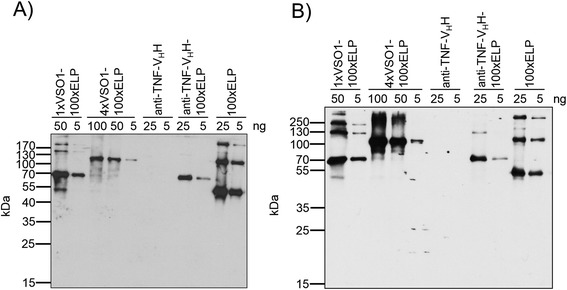
**Immunoblots for the characterization of the capability for cross-reaction of antigens with serum from other spidroins.** Both spidroin-based biopolymers, 1xVSO1-100xELP and 4xVSO1-100xELP, and the control proteins, anti-TNF-V_H_H, anti-TNF-V_H_H-100xELP and 100xELP, were separated using 12% SDS-PAGE, electrotransferred and detected with **A)** mouse serum 1 or **B)** mouse serum 5.

The use of spider silk variants for the production of protein scaffolds for tissue engineering or for engineering of drug delivery systems requires spidroin-based derivatives that do not induce immune responses. Natural spider silks do not cause an inflammatory response [36]. A low immunogenicity for 100xELP is anticipated [28], and has been shown experimentally by studying the T-cell response [32]. In this report, we ask the question whether different-sized fusion proteins of synthetic spidroins and ELP can induce a humoral immune response and if the increasing spidroin content in addition to the higher molecular weight has an influence. For the determination of the immunogenicity elicited by heterologous synthetic spidroin-based biopolymers one group of C57BL/6 J mice (animals 1–4) was immunized with 1xVSO1-100xELP and another (animals 5–8) with 4xVSO1-100xELP. After the fourth immunization, the immune responses in mice were determined by an indirect ELISA against the injected antigen (Figure 3A and B). All sera showed a strong immune response against their antigen, but the antisera raised against the 4xVSO1-100xELP showed binding at higher dilutions, indicating a stronger immune response. Cross-reactivity against the other antigen with a different molecular weight was detected; however, this was very low (Figure 4). We next asked the question if this relative specificity is due to specific structural epitopes formed by the different fusion proteins and we also wanted to explain cross-reactivity. For further immunological analyses, serum 1, which was isolated from a mouse immunized with 1xVSO1-100xELP, and serum 5, which was from a mouse that had been immunized with 4xVSO1-100xELP, were selected. First we analyzed whether the immune response was directed against the spidroin region (VSO1), against the c-myc tag and/or against ELP. For this purpose 1xVSO1-100xELP, 4xVSO1-100xELP, 100xELP and the recombinant protein anti-TNF-V_H_H [37], containing a c-myc tag, were included into the immunoblotting analyses. Additionally, the immune response to the fusion protein anti-TNF-V_H_H-100xELP [37], which also contained the c-myc tag, was analyzed. All proteins were synthesized *in planta*. After developing immunoblots with the appropriate mouse serum, synthetic spidroins were detected, along with anti-TNF-V_H_H-100xELP and 100xELP proteins (Figure 5A and B). Anti-TNF-V_H_H was not recognized by sera isolated from immunized mice, but it was detected by immunoblotting with the anti-c-myc antibody, implicating that the c-myc tag did not induce an immune response (Figure 5A-D). All other proteins mentioned here contained a c-myc tag and were detectable *via* immunoblotting using an anti-c-myc antibody (Figure 5C and D). After incubation with mouse normal serum, no nonspecific reaction was detectable (Figure 5E and F). The relative specificity for the proteins used for immunization could not be seen after immunoblotting. Serum 1 and serum 5 showed comparable binding behavior (Figures 5 and 6). With serum 1 against 1xVSO1-100xELP it was also possible to detect 4xVSO1-100xELP. Analogous antigen-antibody recognition occurred for serum 5 and 1xVSO1-100xELP. The epitopes responsible for relative specificity against the injected proteins in the indirect ELISA are possibly destroyed by denaturation during SDS-PAGE.

**Figure 7:**
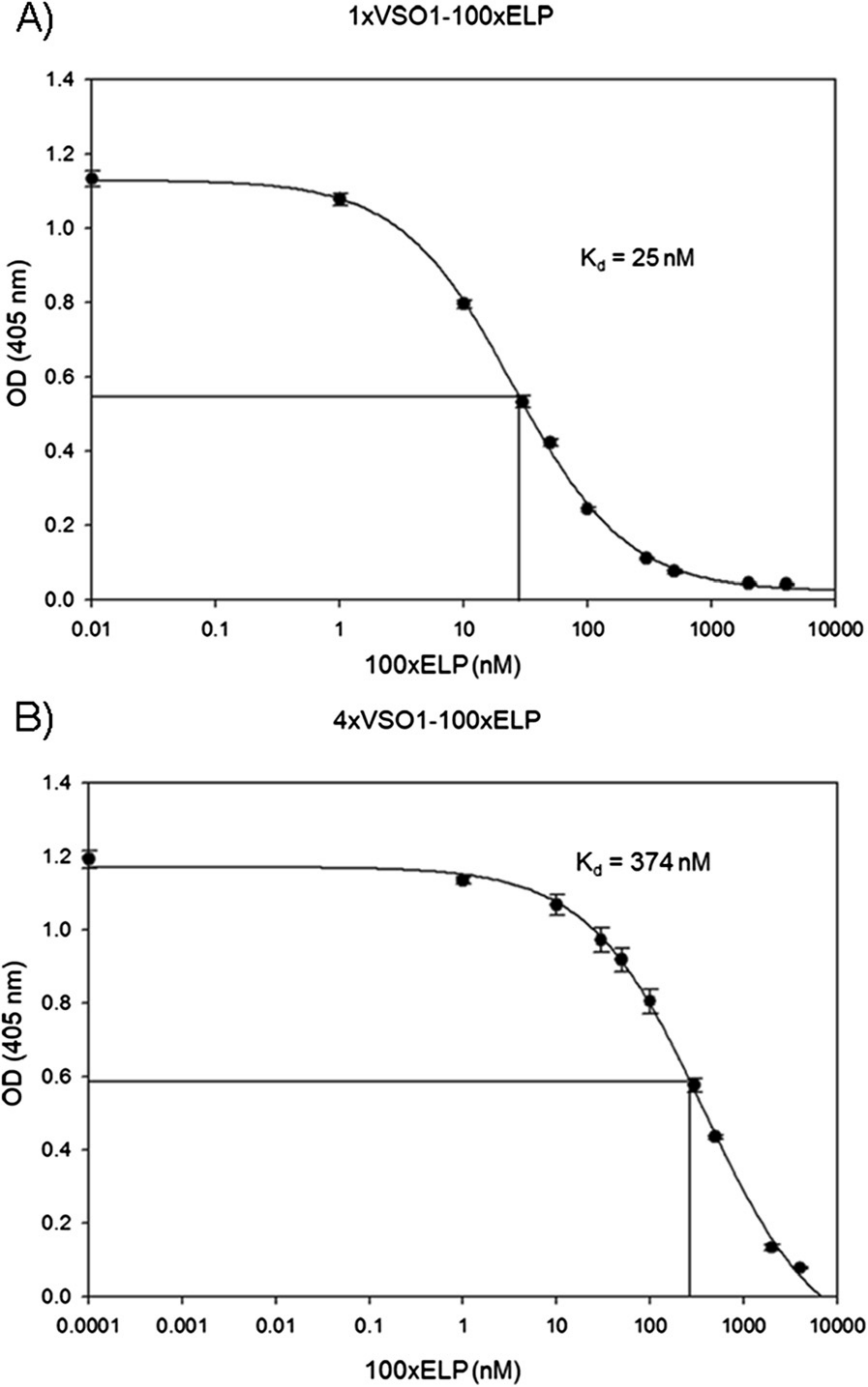
**Characterization of the immune response to ELP by competitive ELISA.** Analyses for a complex consisting of **A)** 1xVSO1-100xELP and mouse serum 1 competing to 100xELP and **B)** 4xVSO1-100xELP, and mouse serum 5 competing to 100xELP.

Additionally, both sera analyzed in more detail showed significant binding to 100xELP in immunoblotting analysis and to anti-TNF-V_H_H-100xELP (Figure 6A and B). Here, we analyzed the binding to ELP in more detail. Polyclonal antibodies are a mixture of different antibodies by nature, with their own respective kinetic characteristics and, in many cases, with differences in epitope specificity. Based on this heterogeneity, the rate constants derived and affinities calculated based on such measurements should be regarded as a mean of different subpopulations. In the case of ELP, the number of different epitopes is limited, because it is a highly repetitive protein consisting of pentamers of very similar if not equal amino acid sequences. Therefore, the complexity is also limited and calculation of a mean dissociation constant *K*_*d*_ is a useful tool to characterize the immune response against ELP-based epitopes. For the selected sera, optimal antigen concentrations were predetermined in dilution analyses and further evaluation of the immunogenicity of ELP was performed by a competitive ELISA. For this purpose, 1xVSO1-100xELP and 4xVSO1-100xELP, respectively, was adsorbed to the polystyrene surface and the binding of the relevant antisera was measured. This binding was inhibited by competition with 100xELP. The calculated mean dissociation constant *K*_*d*_ for the complex 1xVSO1-100xELP-anti 1xVSO1-100xELP from animal 1 and the competitor 100xELP was 25 nM (Figure 7A) and the mean *K*_*d*_ for 4xVSO1-100xELP-anti 4xVSO1-100xELP from animal 5 and 100xELP was 374 nM (Figure 7B). In the immunogenic 1xVSO1-100xELP, the relative ELP content is much higher; thus, in the antiserum a stronger binding (higher affinity) to ELP was induced. However, we cannot exclude that the position of the ELP part in the resulting protein structure of the fusion protein is different in 1xVSO1-100xELP and 4xVSO1-100xELP. The competition curves showed that binding to 1xVSO1-100xELP and to 4xVSO1-100xELP could be completely inhibited (Figure 7). This implicates that the humoral immune response is mainly directed against 100xELP. An overview of all analyzed immune responses is given in Table 3.

**Table 3:** **Overview of analyzed immune responses**

**antibody**	**serum 1**	**serum 5**	**anti-c-myc antibody**	**mouse normal serum**
**antigen (MW)**				
1xVSO1-100xELP (56 kDa)	+	+	+	-
4xVSO1-100xELP (96 kDa)	+	+	+	-
anti-TNF-V_H_H (15 kDa)	-	-	+	-
anti-TNF-V_H_H-100xELP (57 kDa)	+	+	+	-
100xELP (42 kDa)	+	+	+	-

A positive reaction is labeled with a plus sign and no reaction is marked with a bar.

### *In vitro* cytotoxicity assays of the synthetic spidroin-based biopolymers 1xVSO1-100xELP and 4xVSO1-100xELP

**Figure 8:**
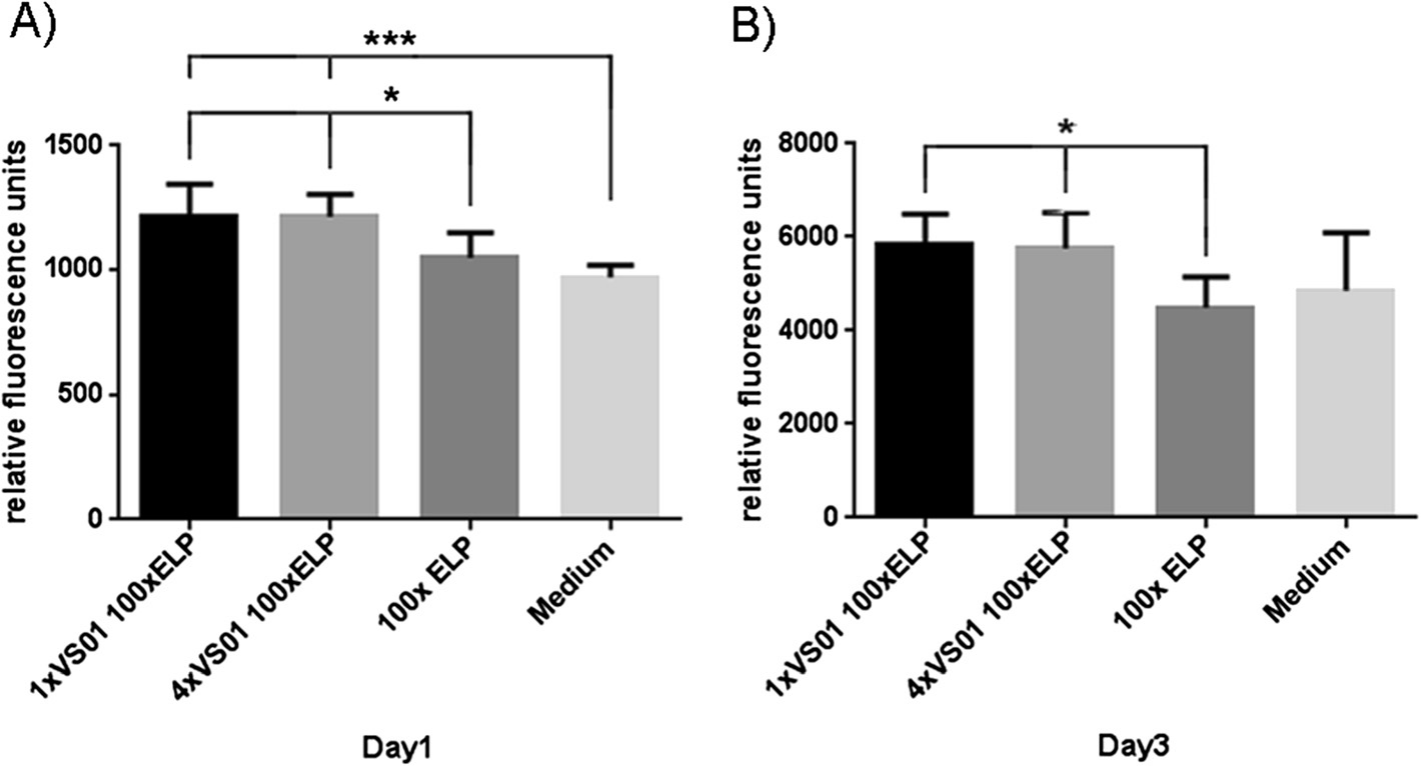
**Metabolic activities of murine embryonic fibroblasts in contact with soluble synthetic biopolymers.** The cells were cultured in medium supplemented with the indicated recombinant protein for 24 hours **(A)** or 72 hours **(B)**. The graphs depict a representative result of three independent repeats (n = 8, mean and SEM).

**Figure 9:**
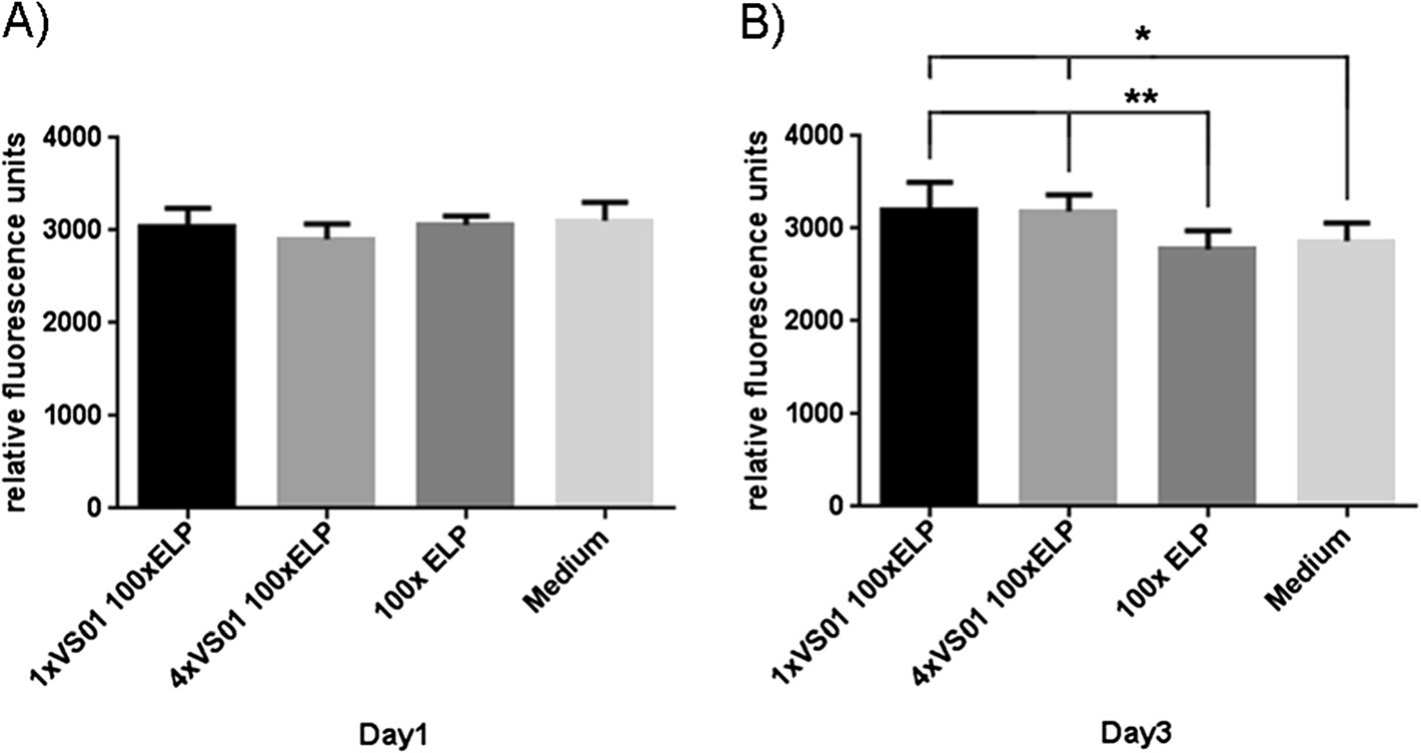
**Metabolic activities of murine embryonic fibroblasts in contact with coated synthetic biopolymers.** The cells were cultured on surfaces coated with the indicated recombinant protein for 24 hours **(A)** or 72 hours **(B)**. The graphs depict a representative result of three independent repeats (n = 8, mean and SEM).

At first, cytotoxicity of the synthetic spidroins fused to ELP was assayed with soluble and coated proteins. In general, cell metabolic activity is determined as an indirect measure for cell vitality and proliferation. This is done by adding a compound to the culture medium, which is metabolized by the living cells to a fluorescent end product. Here, the influence of a recombinant spider silk protein on the metabolism of murine embryonic fibroblasts was tested by adding the protein to the cell culture medium or by coating the culture surface. In both cases an influence could be observed. 1xVSO1-100xELP and 4xVSO1-100xELP significantly stimulated the cells when metabolic activity was determined 24 hours after the addition of the protein (Figure 8A). The same trend was observed on day three; however, now the only statistically significant differences were between 100xELP and 1xVSO1-100xELP and 4xVSO1-100xELP, respectively (Figure 8B). The results for cells grown on protein-coated surfaces were similar (Figure 9A and B), although a significant effect was detectable later (Figure 9B).

**Figure 10:**
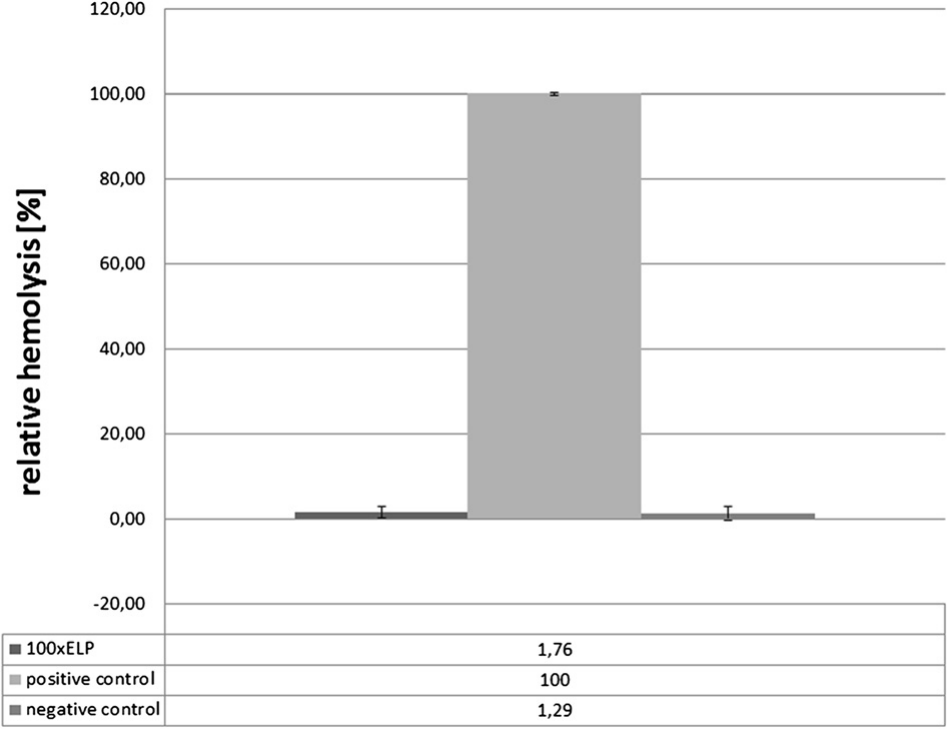
**Hemolysis of human erythrocytes incubated with 100xELP.** The biopolymer was coated on glass coverslips. Free Hb was determined and expressed as percent of the positive control (n = 3, mean and SEM).

In addition, a surface for hemocompatibility of the fusion protein 100xELP was evaluated by direct hemolysis testing. The possible destruction of the erythrocyte membrane was measured by determining the amount of free plasma hemoglobin (Hb). The results showed that 100xELP was not hemolytic when covered on glass coverslips. While 100% relative hemolysis could be achieved in the samples containing catheters as a positive control the relative hemolysis observed in the samples with protein-coated glass coverslips were in the same range as the samples obtained by testing blood on high density polyethylene films used as the negative reference material (Figure 10). In general, the results of these cytotoxicity analyses examining cytotoxicity and hemocompatibility provided a positive preliminary indication for the use of the investigated synthetic spidroin-based biopolymers in medical applications.

## Discussion

The high molecular weight of spider silk proteins is assumed to be a key factor that underpins their outstanding mechanical properties, because all characterized native spider silks consist of proteins with high molecular weights [13]. In this study, we analyzed the relation of the spidroin content in recombinant spidroin-based biopolymers and the corresponding mechanical properties. The elastic penetration modulus E was found to range from 2.7 GPa for 1xVSO1-100xELP to 4.5 GPa for 4xVSO1-100xELP; the synthetic biopolymer with the highest molecular weight that was used in this study. The significant increase of the elastic penetration modulus E, which correlates in a first approximation with the Young’s modulus, appears to be due to the increase in the spidroin content. E values were also comparable to ELPylated MaSp1 derivatives, which were previously investigated [27]. One must take into account that the recombinant spidroin-based biopolymers analyzed here has approximately one-third of the molecular weight of the native MaSp1 and that protein layers were examined. In comparison, for native dragline silk a Young’s modulus of approximately 12 GPa was determined [20]. Additionally, the mechanical characterization of a fiber made from native-sized recombinant spider silk protein showed a Young’s modulus of 21 ± 4 GPa; however, in the same study it was also noted that proteins of lower molecular weight did not yield similar material properties [20]. Therefore, for further intended projects the production of native-sized spidroin containing biopolymers is commendable and has already been successfully performed *in planta* with an intein-based post-translational protein multimerization technology [38].

Low immunogenicity is anticipated for natural spider silks; this feature was exemplarily proven for major ampullate dragline silk collected from *Nephila clavipes* [39]. Here, we presented the first results of a specific antibody response to a spidroin-based biopolymer, which was enabled by a collection of various mouse sera that were prepared after immunization of mice with soluble spidroin-ELP fusion proteins. The spidroin-based proteins additionally contain the c-myc tag. An immune response against the c-myc-tag could not be detected by immunoblotting. All the antigens used in the immunological analyses were produced in plants to allow comparisons in all experiments. The data found by indirect ELISA showed a relative specific reaction against the immunogen, either 1xVSO1-100xELP or 4xVSO1-100xELP. This data provides no insight into whether these specific antibodies in the sera bind to ELP- or spidroin-based epitopes or new overall structures in the different fusion proteins. After denaturation of the antigens and SDS-PAGE, the specific reactions were not observed in the immunoblotting experiments. Competitive ELISA data showed clearly that binding is completely inhibited by 100xELP. We conclude that the humoral immune response against the spidroin-based polymers is directed against epitopes involving 100xELP. The sera raised against 1xVSO1-100xELP bind to 100xELP with a > 15-fold higher mean dissociation constant. These differences in the affinity against ELP support the view (see above) that 1xVSO1-100xELP and 4xVSO1-100xELP induced antibodies against different epitopes. These antibodies with higher mean affinity could be induced either by the higher ELP proportion in the fusion protein compared with 4xSO1-100xELP or by specific structures occurring in the 1xVSO1-100xELP fusion protein. We conclude that, in general, spidroin-based biopolymer variants have to be tested according their immunogenicity in each single case. The structure of fusion proteins could lead to the induction of several new immune responses even if standard basic sequences are used.

Cytocompatibility is a prerequisite for the biomedical application of a material. Therefore, *in vitro* cytotoxicity assays are typically one of the first assessments carried out in the biological evaluation of biopolymers. Recombinant spider silk proteins have been tested in a number of different settings. In previous studies, it was shown that a plant-produced synthetic spidroin derived from MaSp1 and fused with hundred repeats of elastin-like peptides (ELP) resulted in a non-cytotoxic biopolymer that supported the proliferation of mammalian cells [26]. An example of a recombinant spider silk protein consisting of five glycine-rich segments alternating with four polyalanine stretches connected to a non-repetitive globular C-terminal domain by a serine- and alanine-rich linker produced in *E. coli* was tested with primary human fibroblasts. The cells attached to the material and grew on different matrices such as meshes and foams [40]. 3T3 fibroblasts adhered to porous scaffolds of recombinant protein based on spidroin 1, even filling the deeper layers after 14 days [41]. However, a recombinant protein based on the silk of the European garden spider prevented adhesion and cell proliferation of BALB/3 T3 fibroblasts when coated on silicone surfaces [42]. Here, we found a positive stimulation of cellular metabolism, which indicates higher growth rates. This effect was dependent on the composition of the protein. Interestingly, we could even enhance the positive effect when the proteins were freely available in the medium. Although, we cannot rule out that this effect is based on the nutritive value of the proteins, this is rather unlikely owing to the negligible concentration of the protein present.

Biomaterials in contact with blood must show good hemocompatibility, which is often improved by coating. Sulfated silkworm fibroin has been used for this purpose in a study to enhance the hemocompatibility of poly(lactic-co-glycolic) acid vascular grafts [43]. Nevertheless, it has been discussed that larvae from two pyralid moths express silk proteins in their guts, their fat body and their hemocytes. It is assumed that these proteins also take part in immunity and coagulation [44]. We observed no hemolytic effect in the examined samples, indicating that they may serve as biocompatible coatings of blood-exposed implants.

Synthetic spidroins as biomaterials that are produced in biotechnology processes have potential use in a wide range of biomedical material applications. Prominent examples are scaffolds for tissue engineering (films, sponges, hydrogels) and drug delivery systems that trigger an effective immune response after vaccination with particle bound antigens [45,46]. In this study, we partially worked with casted proteins. Since the surface of the resulting films showed a very low roughness, applications as a coating or a wound dressing device is conceivable. Finally, the molecular weight of spidroin-based biopolymers did not influence the cytocompatibility of the casted films.

## Conclusion

The main goal of the present study was the assessment of the immunological properties and cytotoxic effects of synthetic spidroin-based fusion proteins expressed *in planta*. Considering the rising elastic penetration modulus determined by AFM-based nanoindentation with increasing spidroin content, we assume additionally a first relationship between spidroin size and mechanical properties. All available antibody detection systems were used to determine epitope regions, including detection of the c-myc tag, characterization of mouse sera after immunization with the synthetic spidroin-based biopolymers and performance of competitive ELISA with the competitor ELP. Furthermore, analyses of cross-reactivity experiments gave no hint of an immunogenic region in the synthetic spidroin part of the fusion constructs. In the end, cytocompatibility studies provided no indication of spidroin-derived cytotoxicity. This implies that these plant-derived synthetic biopolymers are suitable for use as biomaterials.

## Methods

### Design of plasmids

Synthetic *1xVSO1* was produced by Geneart (Life Technologies, CA, USA) and contained repetitive gene motives from *Nephila clavipes* cDNA [GenBank: M37137.2]. In the course of the synthesis, the codon usage of synthetic *1xVSO1* was adapted to *N. tabacum*. After restriction digest with *Bam*HI and *Bgl*II the gene fragment was ligated into the vector 100xELP-pRTRA [47]. Further insertion of synthetic genes was facilitated with an additional restriction digest of the vector (VSO1)_n_-100xELP-pRTRA with *Bam*HI and ligation of a *Bam*HI/*Bgl*II digested *1xVSO1* gene fragment. After ligation, a functional *Bam*HI restriction site was retained at the 5′-prime end of the synthetic gene. The resulting plasmid (VSO1)_n_-100xELP-pRTRA contained a plant expression cassette consisting of the Cauliflower Mosaic Virus (CaMV) 35S promoter [48], the legumin B4 signal peptide (LeB4) [29], the synthetic gene (*VSO1*)_n_, a c-myc tag [49], 100 repeats of the fusion protein ELP [26] and the ER retention signal KDEL [50]. The expression cassettes were excised with the restriction enzyme *Hin*dIII and inserted individually into the binary vector pCB301-Kan [51], resulting in the plant expression vectors (VSO1)_n_-100xELP-pCB301-Kan.

### Production of plant-expressed synthetic spidroin-based fusion proteins

The binary vectors were transformed into the *A. tumefaciens* strain C58C1 (pGV2260) [52] by electroporation. For stable transformation of tobacco (*N. tabacum* cv. SNN), the leaf disc transformation method reported by Horsch *et al*. [53] was performed. The transgenic plants were cultured on Murashige-Skoog agar containing 50 mg/L kanamycin and analyzed by immunoblotting using an anti-c-myc antibody [54]. High expressing plants were cropped into soil and grown in a greenhouse for 4 to 6 weeks prior to harvesting the leaves. The fusion of the synthetic spider silks to 100xELP enabled protein purification *via* membrane-based inverse transition cycling (mITC) [34]. Therefore, frozen leaf material (−80°C) was crushed, added to preheated (85°C) 50 mM Tris–HCl (pH 8.0) and cooked for 1 hour. Further purification and desalting was performed as described [27]. For determining protein weight and storage, the purified proteins (VSO1)_n_-100xELP were lyophilized (ALPHA2-4LSD; Christ, Osterode, Germany).

### SDS-PAGE and immunoblotting analysis

For analysis of transgenic plants, leaf material was ground in liquid nitrogen. Sample buffer (72 mM Tris, 10% v/v glycerol, 2% w/v SDS, 5% w/v 2-mercaptoethanol and 0.0025 mM bromphenol blue, pH 6.8) was added and the homogenate was incubated for 10 min at 95°C. After centrifugation (30 min, 4°C, 12,000 rpm), the extract (supernatant) was kept and the protein concentration was determined by Bradford assay (Bio-Rad, Germany). Plant extracts or purified proteins, which were also analyzed by immunoblotting, were separated on reducing SDS-PAGE and electroblotted to a nitrocellulose membrane (Whatman GmbH, GE Healthcare, Germany) using 25 mM Tris, 0.1% w/v SDS, 192 mM glycine and 20% v/v methanol. For detection of the transgenic product, the membranes were blocked for 2 hours in 5% w/v fat-free dry milk dissolved in 180 mM NaCl and 20 mM Tris, pH 7.8. The primary antibody was either an anti-c-myc (9E10) supernatant [54] or an anti-(VSO1)_n_-100xELP mouse serum. Therefore, two groups of four mice each (C57BL/6 J) were immunized with 1xVSO1-100xELP or 4xVSO1-100xELP. For the first immunization, 50 μg antigen and complete Freund’s adjuvants (Difco, USA) were used. In the following three immunizations animals were boosted with 20 μg antigen and incomplete Freund’s adjuvants (Difco, USA). Titers from blood samples were evaluated by ELISA and sera were collected one week after the fourth immunization. For immunoblotting analysis the secondary antibody used was a horseradish peroxidase- (HRP-) conjugated anti-mouse IgG from sheep (GE Healthcare UK Ltd., UK). Synthetic spidroin-based fusion proteins were detected by ECL (Amersham ECL Plus TM, GE Healthcare UK Ltd., UK).

### Mechanical Testing of (VSO1)_n_-100xELP

Protein layers for AFM imaging and AFM-based nanoindentation were casted by the drop to drop technique. Therefore, proteins were solubilized in water to a concentration of 1 mg/mL, successively dropped in 20 μL droplets onto glass slides and dried in a vacuum (Vacuum Concentrator 5301; Eppendorf, Germany) at room temperature until achieving layers of required thickness [27]. Measurements of protein layer thicknesses and topographical imaging were performed by an atomic force microscope Nanowizard®II (JPK Instruments, Germany) using either the Contact Mode with an MLCT silicon nitride cantilever (Bruker Cooperation, USA) or silicon cantilevers PPP-NCHR (NANOSENSORS™, NanoWorld AG, Switzerland) with tip radii below 7 nm for the Intermittent Contact Mode. Based on topographical information, roughness data were evaluated. AFM-based nanoindentation was performed to assess the elastic penetration modulus E. For that reason, the same AFM instrument was used to record and evaluate load penetration curves according to an advanced Hertzian model for spherical indenter geometry [27,55] as a course of the load dependent on a penetration depth between 10 to 15 nm. E-values were calculated from a large series of 1225 indentations, which were performed for each protein layer with an orthogonal and lateral inter-sampling point distance of 715 nm and this enabled the statistically evident calculation of E for each material (Additional file 1). Elastic penetration moduli E were calculated from the recorded load penetration curves. Here, a diamond-coated cantilever DT-NCHR #1 (NanoWorld AG, Switzerland) calibrated by the Thermal Noise Method was used. The exact geometry of the diamond-coated cantilever tips was checked by scanning electron microscopy (SEM) before and after indentation measurements.

### Indirect and competitive ELISA

For evaluation of the antibody titer against the specific antigen and to examine the cross-reaction, an indirect ELISA was performed. Ninety-six-well plates (MaxiSorp™ Surface, Thermo Scientific Nunc A/S, Denmark) were coated overnight at room temperature with 500 ng of synthetic spidroin dissolved in 100 μL phosphate-buffered saline for phages (PPBS; 32 mM Na_2_HPO_4_ × 2 H_2_O, 17 mM NaH_2_PO_4_ × H_2_O, 100 mM NaCl, pH 7.2). Bovine serum albumin (BSA, 3% w/v) in phosphate-buffered saline (PBS; 8 mM Na_2_HPO_4_ × 2 H_2_O, 2 mM KH_2_PO_4_, 150 mM NaCl) supplemented with 0.05% (v/v) Tween-20 (PBS-T) was used as the negative control. Blocking was done with 130 μL/well using 3% (w/v) BSA in PBS-T (pH 7.6) for 2 hours at room temperature. Mouse sera were diluted with 3% BSA in PBS-T as indicated in the results section and applied in triplicate using a volume of 100 μL/well on the coated plates for 1.5 hours at 25°C. After five washing steps with PBS-T, a goat anti-mouse IgG conjugated with alkaline phosphatase (Sigma, USA) was added in a dilution of 1:2,000 in 1% (w/v) BSA-PBS-T. Plates were incubated for 1 hour at 25°C followed by five washing cycles with PBS-T. Bound antibodies were detected after the addition of the substrate p-nitrophenyl phosphate (1 mg/mL in 0.1 M diethanolamine-HCl, pH 9.8). The reaction was incubated at 37°C and the absorbance was measured at 405 nm within of 1 hour of the reaction being initiated.

For the competitive ELISA, antigens were dissolved in PPBS and 50 ng/well (1xVSO1-100xELP) or 100 ng/well (4xVSO1-100xELP), respectively, were coated to the microtiter plates. Blocking and washing were performed as mentioned above. Various concentrations of the competitor 100xELP (1 nmol to 4 μmol and without ELP) were premixed for 30 min at room temperature with either a 1:7,500 dilution of mouse serum 1 (against 1xVSO1-100xELP) or a 1:4,000 dilution of mouse serum 5 (against 4xVSO1-100xELP). Both competition partners were diluted in 3% (w/v) BSA in PBS-T. This premix was added in quintuplicates to the coated plates followed by incubation at 25°C for 1.5 hours; 3% BSA in PBS-T was used as negative control. Further processing of the assay was performed as described above.

### Coating of the cell culture material for cytotoxicity tests

The synthetic spidroin-based biopolymers were diluted to a concentration of 50 μg/mL with phosphate-buffered saline without calcium and magnesium (PBS, Life Technologies). Glass coverslips for direct hemolysis assay, coated on both sides, and black microtiter plates for the determination of cell metabolism were coated with the protein by applying the protein solution at 4°C overnight. The liquid was then removed and cell culture plates were dried at room temperature. The coated materials were kept at 4°C or used immediately. PBS without the biopolymer was used as a control.

### Cell metabolism assay

Murine embryonic fibroblasts were obtained from the ATCC (ATCC® SCRC-1045™) and cultured in Dulbecco’s Modified Eagle’s Medium (DMEM) high-glucose (Biochrom, Berlin Germany) with 1% penicillin/streptomycin (Biochrom, Berlin Germany), 1% sodium pyruvate (Biochrom, Berlin, Germany) and 10% fetal bovine serum (Biochrom, Berlin, Germany). The cultures were split thrice weekly and kept in a humidified atmosphere at 37°C and 5% CO_2_. For the measurements, the cells were diluted to 5 × 10^4^ cells/mL and seeded onto microtiter plates. All test samples were done either on surface-coated wells as indicated above or the protein was added to a final concentration of 50 μg/mL to the culture medium. After the indicated time points, 20 μL of CellTiter-Blue® solution (Promega, USA) was added to each well and incubated at 37°C for 2 hours. Fluorescent resorufin was measured by using 560 nm excitation and 590 nm emission filters. All tests were repeated at two independent times and performed at octuplicates. The data were analyzed by one-way ANOVA followed by Tukey’s multiple comparisons test.

### Preparation of blood

The blood was obtained from the Institut für Transfusionsmedizin, Medizinische Hochschule Hannover. The blood donors subscribed a declaration, that they agree that small amounts of their blood could be used for research proposals. Human blood was collected in S-Monovette tubes (Sarstedt, Germany) with citrate and used within 4 hours after collection by pooling equal amounts of blood. The Hb value of the pooled blood was measured and the blood was diluted with PBS to a total blood Hb concentration of 10 ± 1 mg/mL.

### Direct hemolysis assay

The samples were covered with PBS resulting in a surface-to-PBS ratio of 3 cm^2^/mL in screw-cap polypropylene tubes. Subsequently, pooled blood was added at a ratio of 1:7 and incubated at 37°C for 3 hours. During this time, the tubes were inverted carefully twice every 30 minutes. A sample of 1.8 ml was removed from the test tubes and centrifuged at 700–800 g for 15 minutes. Drabkin’s solution (Sigma, Germany) was added to the samples at equal volumes and incubated at room temperature for 15 minutes. Two aliquots of each sample were transferred to a microtiter plate and the absorbance was measured at 540 nm. All measurements were done in triplicates and catheters (ARROWg + ard Blue, Arrow international) and high density polyethylene films (RM-C, Hatano Research Institute) were used as hemolytic and non-hemolytic controls, respectively.

## Additional file

Additional file 1:
**Statistical distribution of elastic penetration moduli E for layers of synthetic biopolymers.** Load penetration curves (n = 1225) were determined per protein sample layer with a thickness of at least 1 μm and a mean surface roughness smaller than 2 nm for a 2.5 × 2.5 μm^2^ grid.
