# Supplementary figures and images for: In planta production of ELPylated spidroin-based proteins results in non-cytotoxic biopolymers
Source: BMC Biotechnol. 2015 Feb 19;15(1):9. doi: 10.1186/s12896-015-0123-2 (PMC4343268; doi:10.1186/s12896-015-0123-2)

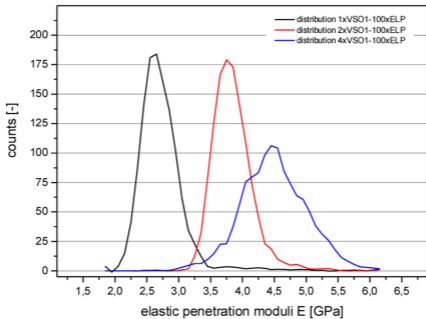

Supplement: Additional file 1: — Statistical distribution of elastic penetration moduli E for layers of synthetic biopolymers. Load penetration curves (n = 1225) were determined per protein sample layer with a thickness of at least 1 μm and a mean surface roughness smaller than 2 nm for a 2.5 × 2.5 μm2 grid. [file 12896_2015_123_MOESM1_ESM.pdf]
